# Mechanisms of action and resistance prevention of synergistic thymol and carvacrol combinations with antibiotics in* Staphylococcus aureus *and* Acinetobacter baumannii*

**DOI:** 10.1007/s13659-025-00518-7

**Published:** 2025-06-06

**Authors:** Cristina Gan, Elisa Langa, Gang Wang, Françoise Van Bambeke, Diego Ballestero, María Rosa Pino-Otín

**Affiliations:** 1 Universidad San Jorge. Campus Universitario Villanueva de Gállego Autovía A, 23 Zaragoza-Huesca, Km. 510, 50830 Villanueva de Gállego, Saragossa Spain; 2 Louvain Drug Research Institute, Université Catholique de Louvain, Brussels, Belgium

**Keywords:** Thymol, Carvacrol, *Staphylococcus aureus*, *Acinetobacter baumannii*, Antibiotic resistance

## Abstract

The use of natural products as antibiotic adjuvants to enhance efficacy and mitigate resistance is increasingly recognized as a promising strategy. This study explored five novel synergistic antimicrobial combinations (SACs) of carvacrol (CARV) and three already identified SACs of thymol (THY) with chloramphenicol, gentamicin, and streptomycin against *Staphylococcus aureus* and *Acinetobacter baumannii*, critical WHO-listed pathogens, and investigated their mechanisms of action and resistance-prevention capabilities. Despite being isomers, CARV and THY exhibited distinct synergistic effects and fractional inhibitory concentration index (FICI) values depending on the antibiotic and bacterial species. The SACs significantly reduced the required antibiotic dose by 4- to 16-fold, with FICI values ranging from 0.25 to 0.5. Growth kinetics revealed that SACs completely inhibited planktonic bacterial growth, outperforming antibiotics alone. Additionally, the SACs demonstrated efficacy in both inhibiting and eradicating biofilms of *S. aureus* and *A. baumannii*. Resistance development studies highlighted that neither THY nor CARV induced resistance in these pathogens. Moreover, SACs combining aminoglycosides with THY reduced the emergence of resistance in *A. baumannii* by up to 32-fold. In *S. aureus*, THY mitigated gentamicin resistance by 16-fold. CARV exhibited similar, albeit slightly less potent, effects.

Mechanistic investigations revealed that THY and CARV exert antimicrobial action by multiple mechanisms, including bacterial membrane depolarization and disruption, efflux pump inhibition, disrupting ATP metabolism and mitigating oxidative stress induced by antibiotics. These findings highlight the potential of SACs to enhance antibiotic efficacy while preventing resistance, positioning them as strong candidates for innovative antimicrobial therapies against multidrug-resistant pathogens.

**Graphical Abstract:**

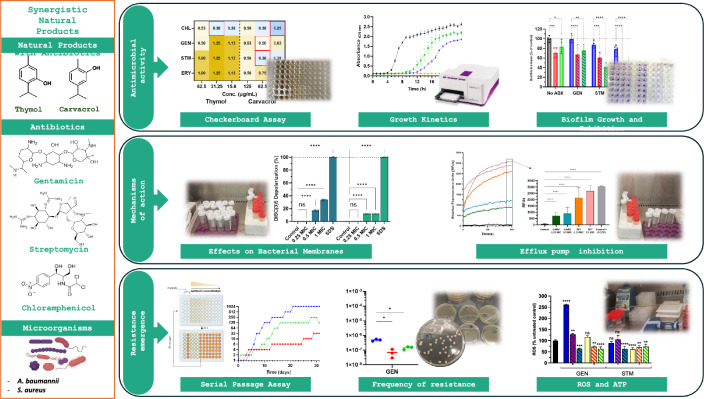

## Introduction

Antibiotic (ABX) resistance poses a significant threat to global health, reducing the effectiveness of treatments for bacterial infections and leading to prolonged illness, higher medical costs, and increased mortality [1]. The emergence of resistant pathogens highlights the urgent need for innovative antimicrobial strategies. Natural products, like plant extracts, are gaining substantial interest as alternative sources [2]. However, plant extracts and their pure natural products present certain limitations: they tend to be more effective against Gram-positive than Gram-negative bacteria, often require high doses to achieve antimicrobial effects comparable to commercial ABXs, and their action is generally less specific [3, 4]. The synergistic interaction between plant phytochemicals and conventional ABXs presents an optimal alternative, combining the efficacy of ABXs with the properties of natural compounds [5]. This approach may extend the lifespan of existing ABXs, which are costly to develop and remain essential tools against infections. Moreover, the combined effects with natural products not only provide an opportunity to lower the minimum inhibitory concentration (MIC) of the ABX, potentially reducing resistance by minimizing ABX use [6, 7], but also take advantage of the broad and nonspecific mechanisms of action of natural products, which make them less prone to resistance development [4, 8].

Natural compounds, such as thymol (THY) and carvacrol (CARV), have emerged as promising candidates due to their antimicrobial properties. THY (2-isopropyl-5-methylphenol) and CARV (2-methyl-5-(1-methylethyl) phenol) are key monoterpenic phenols present in plants from the family Lamiaceae [9]. Chemically, they are monoterpene phenol compounds derived from cymene and are positional structural isomers of each other. As primary components of plant extracts, both THY and CARV have demonstrated antimicrobial activity [9–13]. Moreover, pure THY and CARV are effective against a variety of pathogenic bacteria, including *Escherichia coli* [14, 15], *Staphylococcus aureus*, *Salmonella* [16, 17], *Listeria monocytogenes* [18, 19], *Streptococcus pyogenes* [20] and carbapenem-resistant *Klebsiella pneumoniae* [21].

While research on the combined antimicrobial effects of THY and CARV with commercial ABXs is more limited, recent studies support the potential of these natural compounds as ABX adjuvants. Previous studies have shown that THY exhibited synergistic activity with chloramphenicol (CHL) against *Acinetobacter baumannii*, and with streptomycin (STM) and gentamicin (GEN) against *S. aureus* [22]. Other studies have reported synergistic effects between THY and ABXs such as vancomycin, tetracycline or aminoglycosides against *E. coli* and *S. aureus* [14] and *Klebsiella pneumoniae* [23, 24]. Similarly, CARV has shown synergistic effects with ABX, including clindamycin and penicillin against *S. pyogenes *[20]*,* and with six ABXs, including β-lactams, against multidrug-resistant *Salmonella typhimurium* and *E. coli* [25].

Beyond their effects on planktonic bacteria, studies have also identified synergistic combinations of THY and CARV with ABXs that effectively target bacterial biofilms. For example, Jayakumar et al. explored the synergistic effects of THY combined with ciprofloxacin against *P. aeruginosa,* finding that the combination can significantly reduce planktonic cells and biofilms [26]. Additionally, Miladi et al. found that the combination of THY or CARV with nalidixic acid enhanced the susceptibility of *S. Typhimurium* biofilms [27].

The potential of these natural products not only to enhance ABX efficacy but also to reduce the development of resistance is garnering significant attention. For instance, THY has been shown to reduce resistance when combined with colistin and tetracycline in enterotoxigenic *E. coli* strains. Specifically, it has been observed that THY helps downregulate several virulence and ABX resistance genes [28]. These findings suggest that the use of natural products in synergistic combinations with commercial ABXs could not only enhance ABX efficacy, in both planktonic cells and biofilms, but also mitigate ABX resistance, making them effective tools against resistant pathogens.

This study aims to evaluate the potential of new Synergistic Antimicrobial Combinations (SACs) of THY and CARV with commercial ABXs to enhance antibacterial activity and reduce the emergence of ABX resistance. Specifically, the objectives are as follows: (1) To explore new SACs between CARV and seven widely-used ABXs against eight pathogenic bacteria, and to compare these effects with those of THY; (2) To evaluate the antimicrobial activity of these SACs against these pathogenic bacteria in both planktonic and biofilm forms; (3) To analyze the underlying mechanisms of the observed SACs synergistic effects and (4) To assess whether these products contribute to a reduction in the in vitro generation of ABX resistance.

## Materials and methods

### Antibiotics, adjuvant compounds and reactants

ABXs (a total of seven) tested were selected because they are some of the most widely used ABXs today representing different mechanisms of action. All of them were purchased from Acofarma (Barcelona, Spain) and Sigma- Aldrich (Darmstadt, Germany). Natural products, THY and CARV, were purchased from Sigma- Aldrich (Darmstadt, Germany). Table 1 summarizes the detailed information for each compound.

**Table 1:** Antimicrobial compounds used for the antibacterial tests

Antibiotic/natural product	Abbreviation	Chemical family	CAS number	Purity	Molecular weight (g/mol)	Water solubility (mg/L)
Gentamicin	GEN	Aminoglycosides	1403-6-3	≥ 97%	447.6	12,000 (25 °C)
Streptomycin	STM		57-92-1	≥ 97%	581.6	12,800 (25 °C)
Chloramphenicol	CHL	Amphenicols	56-75-7	97.5%	323.1	2500 (25 °C)
Amoxicillin	AMO	Beta-lactams	26,787-78-0	96–102%	365.4	4000 (25 °C)
Ampicillin	AMP		69-53-4	≥ 90%	394.4	10,000 (21 °C)
Erythromycin	ERY	Macrolides	114-07-8	95.9%	733.9	2000 (25 °C)
Tetracycline chlorhydrate	TC	Tetracyclines	64-75-5	99.2%	444.4	231 (25 °C)
Carvacrol	CARV	Monoterpenes	499-75-2	100%	150.2	1250 (25 °C)
Thymol	THY		89-83-8	100%	150.2	980 (25 °C)

### Bacterial strains and growth conditions

A total of 8 reference bacterial strains were selected for this study, Including both Gram-positive (*Bacillus subtilis* ATCC 6633, *Staphylococcus aureus* ATCC 9144, and *Streptococcus agalactiae* ATCC 12386) and Gram- negative bacteria (*Acinetobacter baumannii* ATCC 19606, *Escherichia coli* ATCC 25922, *Klebsiella aerogenes* ATCC 13048, *Klebsiella pneumoniae* C6, *and Salmonella enterica* ATCC 13311). All microorganisms were obtained as freeze-dried Culti-loops™ bacteria from Thermo Scientific (Dartford, United Kingdom). Upon receipt, the bacteria were rehydrated and stored at − 80 °C in cryovials (Deltalab S.L., Barcelona, Spain) until further use. All procedures related to the handling and cultivation of bacterial strains were performed in accordance with the guidelines provided in the ATCC and Thermo Scientific product sheets for each strain.

### Determination of the antimicrobial activity alone or in combination:

#### Minimum Inhibitory Concentration (MIC) and Checkerboard assay

MIC values were determined using the broth microdilution method following CLSI (M07-A9, 2018) and ISO 207776–1 (2019) guidelines. Bacterial cultures were incubated overnight at 37°C (30°C for *K. aerogenes*) and adjusted to the McFarland standard, CLSI, 2018 (~ 10^5^ CFU/mL). ABX stock solutions were prepared in distilled water, while natural compounds were solubilized in 5% DMSO (CAS: 67-68-5, Fisher Bioreagents, Madrid, Spain). Maximum solvent concentration per well was 1.25%. This concentration was found to be innocuous for all bacterial strains used. Two-fold serial dilutions of ABXs and natural products were made in 96-well microplates, ranging from 2000 to 3.91 µg/mL. A positive control for bacterial growth and a negative control for sterility were included. After incubation at 37°C for 20 h, the MIC was determined as the lowest concentration preventing visible growth, confirmed by measuring absorbance at 625 nm using a Synergy H1 Hybrid Multi-Mode microplate reader (BioTek, USA).

For synergy assessment, a checkerboard assay was performed [25, 29] with natural products (drug A) and ABXs (drug B). Natural products were serially diluted vertically (columns 1–7), and ABXs horizontally (rows A-G), starting at 4X MIC. Plates were inoculated with adjusted bacterial suspension and incubated for 20 h at 37°C. Absorbance (625 nm) was measured to assess bacterial growth. The fractional inhibitory concentration index (FICI) was calculated for each combination using Eq. 1 [29]:

1 $$FICI={FIC}_{A}+{FIC}_{B}=\frac{{MIC}_{A+B}}{{MIC}_{A}}+\frac{{MIC}_{B+A}}{{MIC}_{B}}$$

FIC_A_ represents the MIC of drug A (Natural product) in the presence of drug B (ABX) divided by the MIC of drug A alone. FIC_B_ being MIC of drug B in the presence of drug A divided by the MIC of drug B alone. Based on the European Committee on antimicrobial susceptibility testing guidelines [30], the interpretation of FICI values is as follows: ≤ 0.5 indicates synergy; 0.5 < FICI ≤ 1 additivity; 1 > FICI < 2 no interaction; and ≥ 2 antagonistic effects.

#### Growth kinetics tests

The growth kinetics tests were performed on the five CARV SACs (with FICI ≤ 0.5, highlighted in grey in Table 2) against *A. baumannii* and *S. aureus*, to complement those previously identified with THY [22]. Bacterial samples were adjusted and subjected to sublethal doses of CARV and ABXs, both individually and in combination, based on the checkerboard test results. The experiments were performed in a 96-well microplates, incubated at 37ºC, with absorbance readings taken hourly for 24 h. Each experiment was performed in triplicate. The resulting kinetic curves were analysed using a logistic model for sigmoid microbial growth [31] and fitted to Eq. (2) model with the Excel Solver add-in (Microsoft 365). To characterize the growth curves, the maximum achievable population density (carrying capacity, Cmax), the intrinsic rate of population increase (r), and the time required to reach half of Cmax (tm50) were calculated using the following equation:

**Table 2:** Minimum inhibitory concentrations of commercial antibiotics thymol and carvacrol and fractional inhibitory concentration index of synergistic antimicrobial combinations against various pathogenic microorganisms

Microorganism	Commercial ABX	MIC CARV alone	MIC ABX alone	MIC CARV combination	MIC ABX combination	FIC_I_	ABX MIC fold reduction
*A. baumannii*	CHL	250	62.5	31.3	7.8	**0.25**	**8**
	ERY		15.6	125	1	0.56	16
	GEN		15.6	62.5	3.9	**0.50**^**1**^	**4**
	STM		250	62.5	15.6	**0.375**	**16**
*B. subtilis*	ERY	250	15.6	125	7.8	1	2
*E. coli*	ERY	500	250	500	250	2	1
	GEN		31.3	500	31.3	2	1
	STM		125	500	125	2	1
*K. aerogenes*	CHL	250	31.3	125	7.8	0.75	4
	ERY		62.5	250	62.5	2	1
*K. pneumoniae*	AMO	250	250	250	250	2	1
	AMP		125	250	125	2	1
	ERY		62.5	250	62.5	2	1
*S. agalactiae*	CHL	250	15.6	3.9	7.8	0.52	2
	STM		62.5	31.25	31.25	0.65	2
*S. aureus*	CHL	250	31.25	125	15.6	1^2^	2
	GEN		15.6	62.5	3.9	**0.50**^**3,4**^	**4**
	STM		62.5	62.5	15.6	**0.50**^**5**^	**4**
	TC		62.5	125	31.25	1	2
*S. enterica*	CHL	125	15.6	62.5	7.8	1	2
	ERY		31.3	125	15.6	1.5	2
	STM		31.3	62.5	15.6	1	2

Microorganism*	Commercial ABX	MIC THY alone	MIC ABX alone	MIC THY combination	MIC ABX combination	FIC_I_	ABX MIC fold reduction
A. baumannii^*^	CHL	125	62.5	31.25	7.8	**0.375**	**8**
S. aureus^*^	GEN	250	15.6	31.25	3.9	**0.3756**	**4**
	STM		62.5	62.5	7.8	**0.375**^**7**^	**8**
	Concentration is given in µg/mL; MIC, Minimum Inhibitory Concentrations; ABX, Antibiotic; THY, thymol; CARV, carvacrol; FICI; Fractional Inhibitory Concentration Index; SACs, Synergistic Antimicrobial Combinations ^*^data from Gan et al. 2023 [22] Comparison with bibliographic data: 1. FICI (*A. baumannii* multiresistant isolates) = 0.375 [37] 2. FICI (MSSA) = 0.5 [38] 3. FICI (MRSA strains) = 0.25—0.5 [38] 4. FICI (Reference S. aureus strain) = 0.50 [23] 5. FICI (five MRSA strains) = 0.26—0.5 [38] 6. FICI *( S. aureus* ATCC 12624) = 0.19 [85] 7. FICI (MRSA) = 0.5 [38]						

2 $$\text{Absorbance}=\frac{\text{Cmax}}{1+{e}^{b-rt}}$$

In this equation, t is time (h) and b is a fitting parameter.

To characterize the mechanism of action and resistance of the eight SACs involving THY and CARV with GEN, STM, and CHL against *S. aureus* and *A. baumannii* (grey rows in Table 2), the following experiments were conducted (2.3.3–2.7 sections).

#### Biofilm formation inhibition assay and disruption of preformed biofilms

Biofilm biomass was measured using the crystal violet (CV) staining method [32, 33]. To evaluate the effect of ABX and natural compounds on biofilm biomass production, overnight cultures of *A. baumannii* and *S. aureus* were grown to stationary phase in Nutrient Broth (NB) and Tryptic Soy Broth (TSB), respectively, and adjusted to 10⁶ CFU/mL. Test plates were prepared by adding 100 μL of bacterial suspension and 100 μL of test compounds (ABXs, THY, CARV, or their combinations, all at 0.25 MIC), with a growth positive control included. Plates were incubated at 37 °C for 24 h. After incubation, non-adherent bacteria were removed, wells washed twice with PBS, air-dried, and stained with 1% (w/v) CV for 20 min. Excess CV was removed, wells washed with PBS, air-dried, and biofilm solubilized with 125 μL of 60% (v/v) acetic acid. Absorbance at 570 nm was measured using a microplate reader and data were expressed as a percentage of growth relative to untreated positive controls. The same method was used to assess the ability to reduce biomass of preformed biofilms. Overnight cultures of *A. baumannii* and *S. aureus* were adjusted to 10⁶ CFU/mL, and 100 μL of bacterial suspension and fresh medium were added to wells. Plates were incubated at 37 °C for 24 h to form mature biofilms. Biofilms were then treated with 200 μL of fresh medium containing test compounds (the same treatments as used in the inhibition assay) for an additional 24 h. Untreated wells served as controls. After treatment, wells were processed and biomass was quantified using the CV method, as described in the inhibition assay.

### Mechanisms involved in thymol and carvacrol synergy with antibiotics

#### Outer membrane permeability assay

Outer membrane permeability was assessed using the 1-*N*-phenylnaphthylamine (NPN) uptake assay. NPN is a hydrophobic fluorescent probe that becomes highly fluorescent upon integration into the disrupted outer membrane of Gram-negative bacteria. Overnight bacterial cultures were centrifuged, resuspended in BET phosphate buffer (NaCl 110 mM, KCl 7 mM, NH4Cl 50 mM, Na_2_HPO_4_ 0.4 mM, Tris base 52 mM, Glucose 0.2%, pH 7.5), and adjusted to OD_620_ = 0.1. NPN (10 μM final concentration) was added to the bacterial suspension, and 100 μL of the mixture was dispensed into 96-well plates containing 100 μL of test compounds (THY or CARV at 0.125–1 × MIC). Alexidine (25 μM, Sigma-Aldrich, USA) served as a positive control, and untreated buffer as a negative control. Plates were incubated for 10 min at room temperature, and fluorescence (λexc/λem: 340/410 nm) was measured with a Synergy H1 Microplate Reader.

Outer membrane permeability was calculated using the Eq. 3:

3 $$\left(\text{\%}\right)\text{OM permeability}=\frac{FLUsample -FLUc-}{FLUc+ - FLUc-}\times 100\text{\% }$$

where FLU_sample_ is fluorescence for each condition, FLUc- for buffer alone, and FLUc + for Alexidine, a known bacterial membrane disruptor.

#### Cellular membrane permeability assay

Cellular membrane permeabilization was evaluated using the Propidium Iodide (PI) assay [34]. PI binds to DNA and fluoresces only when both the outer and inner bacterial membranes are permeable, resulting in a 20–30-fold fluorescence increase. Overnight cultures were centrifuged, resuspended in BET buffer and adjusted to OD₆₀₀ = 0.1. Bacterial suspension was mixed with PI (6 μM final concentration) and incubated in darkness for 30 min. In 96-well plates, 50 μL of the PI-bacteria mixture was combined with 50 μL of THY or CARV (MIC to 1/8 MIC) in BET phosphate buffer. Fluorescence (λexc/λem: 540/610 nm) was measured at 25 °C after 15 min. Cellular membrane permeabilization percentage was calculated using Eq. 4.

4 $$(\text{\%})\text{ CM permeability}=\frac{FLUsample -FLUc-}{FLUc+ - FLUc-}\times 100\text{\%}$$

where FLU_sample_ is fluorescence for each condition, FLUc- for buffer alone, and FLUc + for Alexidine, a known bacterial membrane disruptor.

#### Membrane depolarization assay

Membrane depolarization was assessed using the DiSC3(5) assay [35]. DiSC3(5) is a fluorescent lipophilic dye that accumulates in polarized bacterial membranes. When the membrane potential is depolarized, the dye is released, leading to an increase in fluorescence. Overnight cultures of *A. baumannii* and *S. aureus* were centrifuged, washed with PBS, and resuspended in Tris buffer saline (50 mM, pH 7.4) with 2 mM EDTA for *A. baumannii*. After a 5-min incubation and washing, both species were resuspended in Tris buffer saline with 50 mM glucose and adjusted to OD_620_ = 0.5. DiSC3(5) (10 μM final concentration) was added, and 100 μL of this bacterial suspension was mixed with 100 μL of THY or CARV (MIC to 1/8 MIC) in 96-well plates. After 30 min of incubation at 37 ºC in darkness, fluorescence (λexc/λem: 622/670 nm) was measured. Bacterial cells treated with 0.5% SDS served as a positive control, and cells in Tris buffer saline as a negative control. Experiments were performed in triplicate.

#### Efflux pump inhibition assay

The efflux pump inhibition ability of THY and CARV at two concentrations (0.25 and 0.5 MIC) was assessed using the ethidium bromide (EtBr) accumulation assay [36]. EtBr is a fluorescent compound whose fluorescence increases significantly when intercalated into DNA. Efflux pump systems, such as AdeABC in *A. baumannii* actively expel EtBr from bacterial cells. An inhibition of these pumps would result in higher intracellular EtBr concentrations and, consequently, increased fluorescence. Overnight bacterial cultures were centrifuged (4000 rpm, 10 min) and resuspended in PBS to a 0.2 OD at 600 nm. Then, 90 μL of the bacterial suspension were added to each well of a black 96-well plate, along with 100 μL of the test substances. Carbonyl cyanide m-chlorophenylhydrazone (CCCP), a proton motive force uncoupler, was used as a positive control at 1000 μg/mL. To each well, 5 μL of glucose (final concentration 0.2%) and 5 μL of EtBr (final concentration 10 μM) were added. EtBr fluorescence was measured every 15 s for 1200 s (λexc/λem: 535/590 nm). For preincubation experiments, overnight bacterial cultures were centrifuged, resuspended in growth medium with THY or CARV at 0.25 or 0.5 MIC, and preincubated for 6 h. After preincubation, cells were washed twice, resuspended in PBS to 0.2 OD at 600 nm, and the EtBr accumulation assay was performed as described above.

### Multi-step resistance studies: Selection of resistance by serial passage assay

Bacterial resistance development was assessed using serial passage assay. *A. baumannii* and *S. aureus* cultures, adjusted to the McFarland standard, were inoculated in 96-well plates containing serial dilutions of ABXs alone or combined with THY and CARV at synergy concentrations. Plates with THY and CARV alone were also included. The plates were incubated at 37°C for 20 h, and the MIC was determined as described in Sect. 1.3.1. On subsequent days, the culture from the well showing positive growth (½ MIC of the previous day) was readjusted to McFarland and used to inoculate new plates with daily readjustment of ABX concentrations if necessary. This procedure was repeated for 30 days. After the final passage, bacteria were plated on antibiotic-free agar to assess resistance reversion and MIC was calculated by microdilution. MIC ratios (MIC on day *n* [MIC_n_] relative to the initial MIC [MIC_0_], expressed as MICₙ/MIC₀) were plotted over time to monitor resistance development.

### Single-step resistance studies: frequency of resistance (FoR)

Spontaneous mutation frequency was assessed by plating agar with 4X MIC of ABXs, with or without THY and CARV at synergy sub-MIC concentrations. Separate plates containing only THY and CARV (4X MIC) were also prepared. Overnight bacterial cultures of *A. baumannii* and *S. aureus* were adjusted to ~ 10^9^ CFU/mL, and 50 μL of each culture was spread onto the plates. Control plates without ABXs were included. After 48 h of incubation at 37 °C, CFUs were counted. The frequency of resistance (FoR) was calculated as the ratio of colonies on ABX plates to those on control plates.

### Intracellular reactive oxygen species (ROS) and ATP measurement

Intracellular ROS and ATP levels were measured using the ROS Intracellular Assay Kit (OxiSelect™, STA-342; Cell Biolabs, USA) and ATP Determination Kit (A22066; Molecular Probes, Invitrogen, USA). Overnight cultures of *A. baumannii* and *S. aureus* (parent and serial passage generated-resistant strains) were adjusted to OD_600_ = 0.2. Cells were incubated for 1 h at 37 °C with 0.5 MIC of GEN or STM, with or without THY or CARV. After centrifugation (400 rpm, 10 min), cells were resuspended in PBS to OD_600_ = 0.2. For ROS measurement, 90 μL of bacterial suspension and 10 μL of 10 μM DCF-DA were added to each well of a 96-well plate and incubated for 30 min at 37 °C in the dark. Then, fluorescence (λexc/λem: 480/530 nm) was measured. ROS levels were expressed as a percentage of untreated controls.

For ATP measurement, 90 μL of bacterial suspension and 10 μL of ATP standard solution (1.25 μg/mL firefly luciferase, 0.5 mM d-luciferin, 1 mM Dithiothreitol, 25 mM Tricine buffer (pH 7.8), 5 mM MgSO_4_, 0.1 mM EDTA and 0.1 mM Sodium Azide) were added to each well and incubated in the dark at room temperature for 10 min. Luminescence was then measured, and ATP levels were expressed as a percentage relative to control.

## Results and discussion

### Antimicrobial activity of synergistic combinations of thymol, carvacrol, and commercial antibiotics

#### Minimum inhibitory concentration and checkerboard assay

The initial step involved determining the minimum inhibitory concentrations (MICs) of CARV to evaluate their intrinsic antimicrobial activity. This allowed us to subsequently assess potential synergistic interactions with antibiotics by calculating the fractional inhibitory concentration index (FICI), a quantitative metric of combined antimicrobial efficacy.

Table 2 displays the MIC and the FICI values for CARV in combination with various antibiotics across a range of bacterial species. MIC values for CARV alone ranged from 125 to 500 µg/mL, with *S. enterica* being the most susceptible (MIC = 125 µg/mL) and *E. coli* the most resistant (MIC = 500 µg/mL), indicating moderate to low intrinsic antimicrobial activity. Notably, SACs (defined by FICI ≤ 0.5, highlighted in grey) were observed against *A. baumannii* and *S. aureus*. In the case of *A. baumannii*, three SACs were detected (with CHL, GEN, and STM), achieving up to 16-fold reductions in ABX MICs. For *S. aureus*, synergy was observed with GEN and STM, also reducing MICs by fourfold. In contrast, combinations with other bacterial species generally resulted in FICI values ≥ 1, suggesting additive or indifferent effects.

**Fig. 1:**
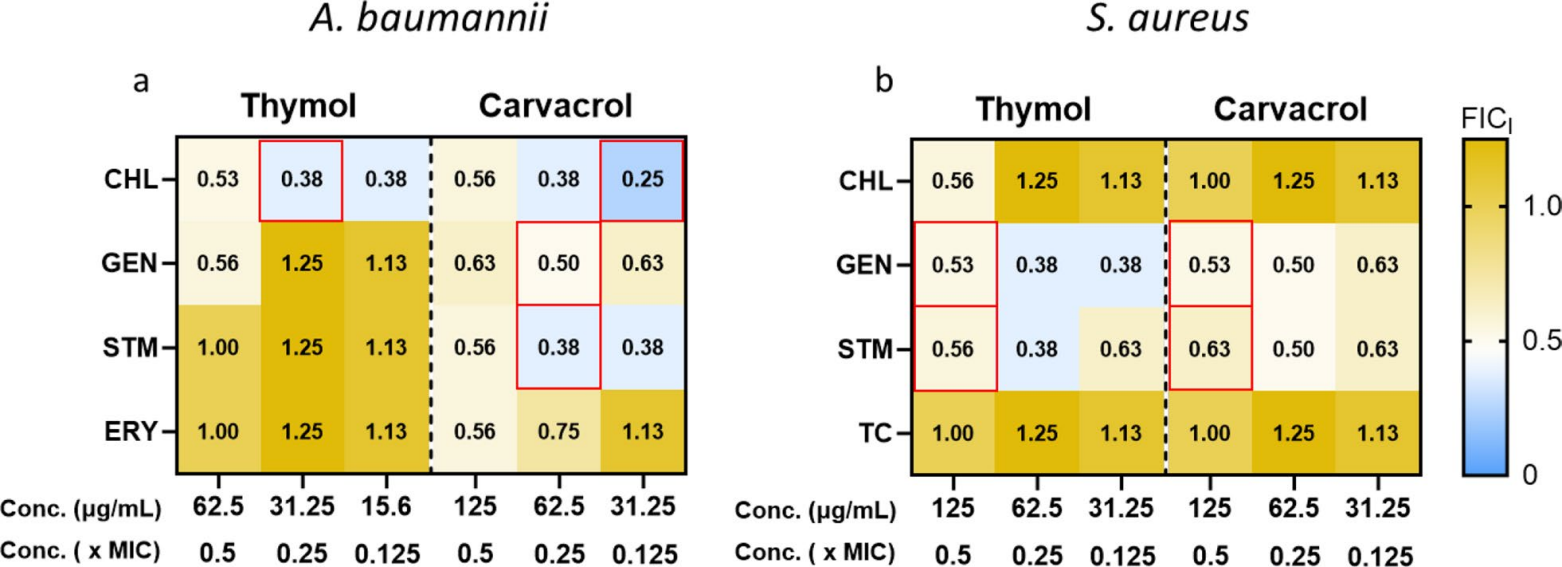
Heatmap plots of the Fractional Inhibitory Concentration Index (FICI) for combinations of thymol (THY) and carvacrol (CARV) with four antibiotics against (**a**) *A. baumannii* and (**b**) *S. aureus*. THY and CARV were tested at three subinhibitory concentrations (0.5×, 0.25×, and 0.125× MIC), indicated at the base of each heatmap as “conc.” (concentration, in µg/mL). Each square displays the corresponding FICI value, calculated using the checkerboard microdilution method. For each combination, FICI values were determined across a two-dimensional matrix of concentration pairs, and the values shown represent those that produced the greatest reduction in antibiotic concentration. Combinations exhibiting the strongest synergy—i.e., the greatest MIC reduction—are outlined in red and were selected for further analysis. The color gradient represents increasing FICI values, ranging from blue (synergy) to yellow (no interaction). A FICI ≤ 0.5 denotes synergistic interaction

When compared with the SACs identified for THY in our previous study (end of Table 2) [22], both similarities and differences in synergy patterns were observed. Although THY and CARV are structural isomers, they exhibited distinct synergistic profiles. For *A. baumannii* (Fig. 1a), both THY and CARV showed synergy with CHL, but only CARV demonstrated additional synergy with GEN and STM. For *S. aureus* (Fig. 1b), both THY and CARV show synergy with GEN and STM. These combinations have recently begun to receive significant attention, as they also appear effective against resistant bacteria isolated from patients. A very recent study investigated the essential oil of *Lippia origanoides* and its main components (including CARV), reporting a FICI of 0.375 for the combination of GEN with CARV against multidrug-resistant *A. baumannii* strains [37]. Another recent study [38] examined the effects of CARV on MRSA strains of *S. aureus* in combination with GEN and STM, reporting FICI values within ranges comparable to our findings (see Table 2).

The fact that the antibiotics involved in the observed synergies—CHL, STM, and GEN—primarily target intracellular components supports further mechanistic investigations into how natural compounds such as CARV and THY may facilitate antibiotic uptake or retention within bacterial cells.

#### Growth kinetics tests

While MIC values provide a static snapshot of bacterial susceptibility at a fixed endpoint, growth kinetics offer a dynamic perspective on antimicrobial activity over time. Moreover, this approach allowed the observation of not only complete growth inhibition but also potential sublethal effects.

**Fig. 2:**
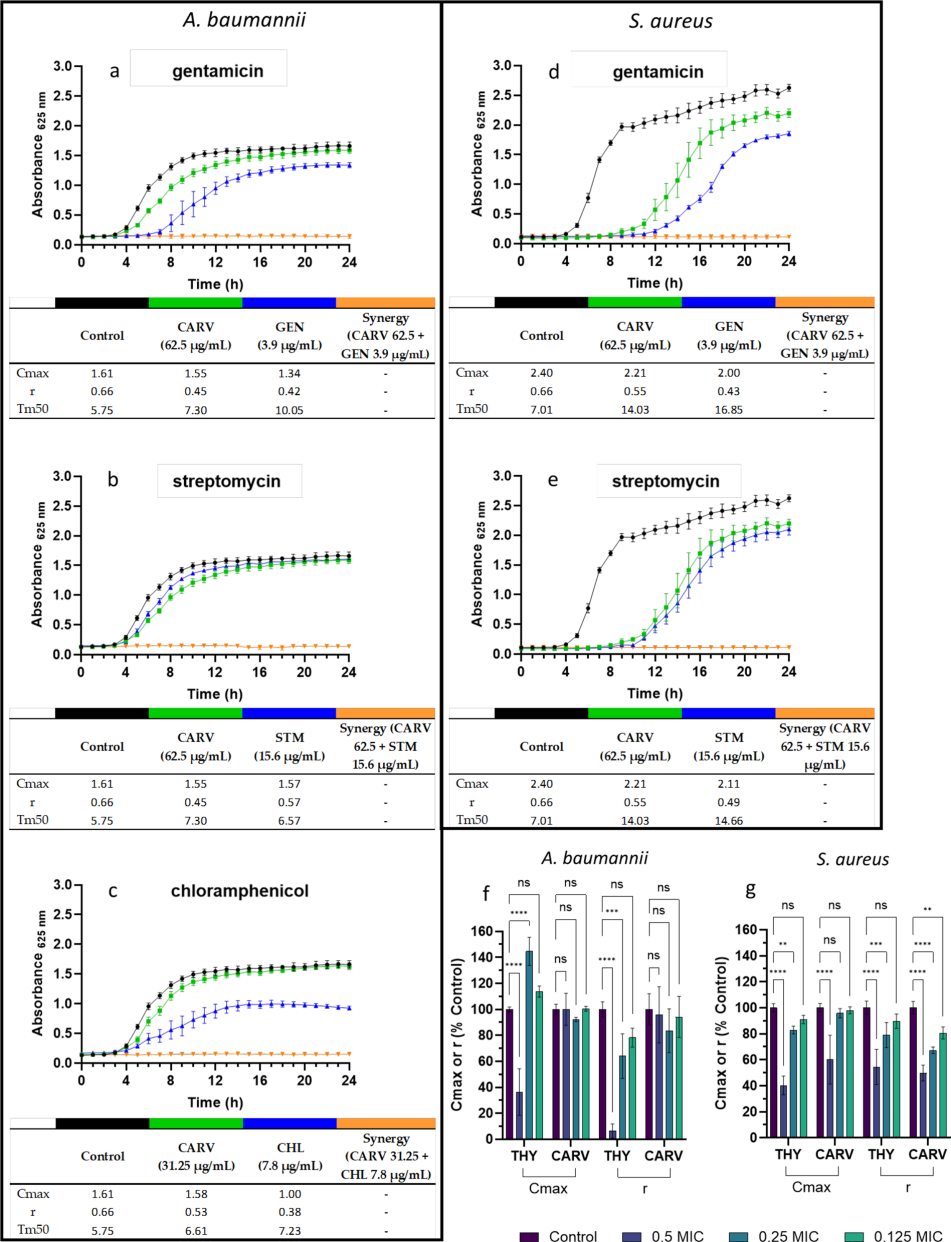
Growth kinetics of *A. baumannii* (**a**–**c**) and *S. aureus* (**d**, **e**) treated with carvacrol (CARV), individual antibiotics (ABXs), and their synergistic combinations at subinhibitory concentrations. Tables below each plot report kinetic parameters: maximum population density (Cmax), growth rate (r), and Tm50 values for each curve. Error bars indicate standard deviations (n = 4). Panels f, g show the relative effects of subinhibitory concentrations of thymol (THY) and CARV on the kinetic parameters of *A. baumannii* and *S. aureus*, expressed as percentage differences from the control. THY data in these panels were sourced from Gan et al. 2023 [22] and are included here to enable direct comparison with CARV. Statistical significance was assessed using two-way ANOVA (****p < 0.0001, ***p < 0.001, **p < 0.01, *p < 0.05, ns = not significant)

Figure 2 shows the growth kinetics of *A. baumannii* and *S. aureus* exposed to the five SACs of CARV, as well as to ABXs and CARV individually, each at the subMIC concentrations used in the checkerboard test. The kinetic profiles reflect the effect of each treatment on bacterial population dynamics. For a more detailed characterization of the growth kinetic profiles, Cmax, r and Tm50 values are presented in a table below the graphs. The ABXs at synergistic concentration have a greater impact on Cmax than CARV, though r values are generally similar across treatments, except for CHL, which has a slower effect compared to CARV. The synergy curves consistently show complete growth inhibition.

Figure 2f and g provide a comparison of the relative effects of THY and CARV on two key kinetic parameters—maximum population density (Cmax) and growth rate (r). These comparisons provide insight into sublethal inhibitory effects that are not captured by MIC alone. The data for THY shown in panels f and g were extracted from a previous study [22] and are presented to enable a direct comparison with the new results obtained for CARV.

In *S. aureus* (panel g), THY demonstrates significantly stronger inhibitory activity than CARV. THY significantly reduces Cmax at both 0.25 and 0.5 MIC, and causes a sharp decrease in r at both concentrations. In contrast, CARV only reduces Cmax at 0.5 MIC, but consistently decreases the growth rate across all three subinhibitory concentrations tested (0.125, 0.25, and 0.5 MIC). In *A. baumannii* (panel f), CARV has minimal to no measurable impact on either Cmax or r at any of the subinhibitory concentrations, in line with the kinetic profiles observed in panels 2a–c. THY significantly reduces both Cmax and r (at 0.5 MIC), indicating strong inhibitory effects.

#### Antimicrobial effect of SACS in biofilm biomass

A bacterial biofilm is a community of bacteria that adhere to a surface and are encased in an extracellular matrix, typically composed of a mixture of polysaccharides, proteins, and extracellular DNA (eDNA) [39]. Biofilm formation is regulated by quorum sensing (QS), a cell-to-cell communication mechanism that synchronizes gene expression in response to population density [40]. This structure provides a physical barrier formed by the extracellular matrix, which hinders the penetration of antimicrobials to the bacteria within [41]. Moreover, bacteria within the biofilm are often in a metabolically slower state, reducing the efficacy of ABXs that rely on active processes, such as protein synthesis.

Biofilm formation is a major factor contributing to antibiotic resistance and the persistence of infections, as these structured bacterial communities are significantly more difficult to eradicate than their planktonic counterparts.

Consequently, the antimicrobial efficacy of SACs should be evaluated not only against planktonic cells but also against biofilms, to assess their ability to both prevent biofilm formation and disrupt mature biofilms formed by *A. baumannii* and *S. aureus*.

**Fig. 3:**
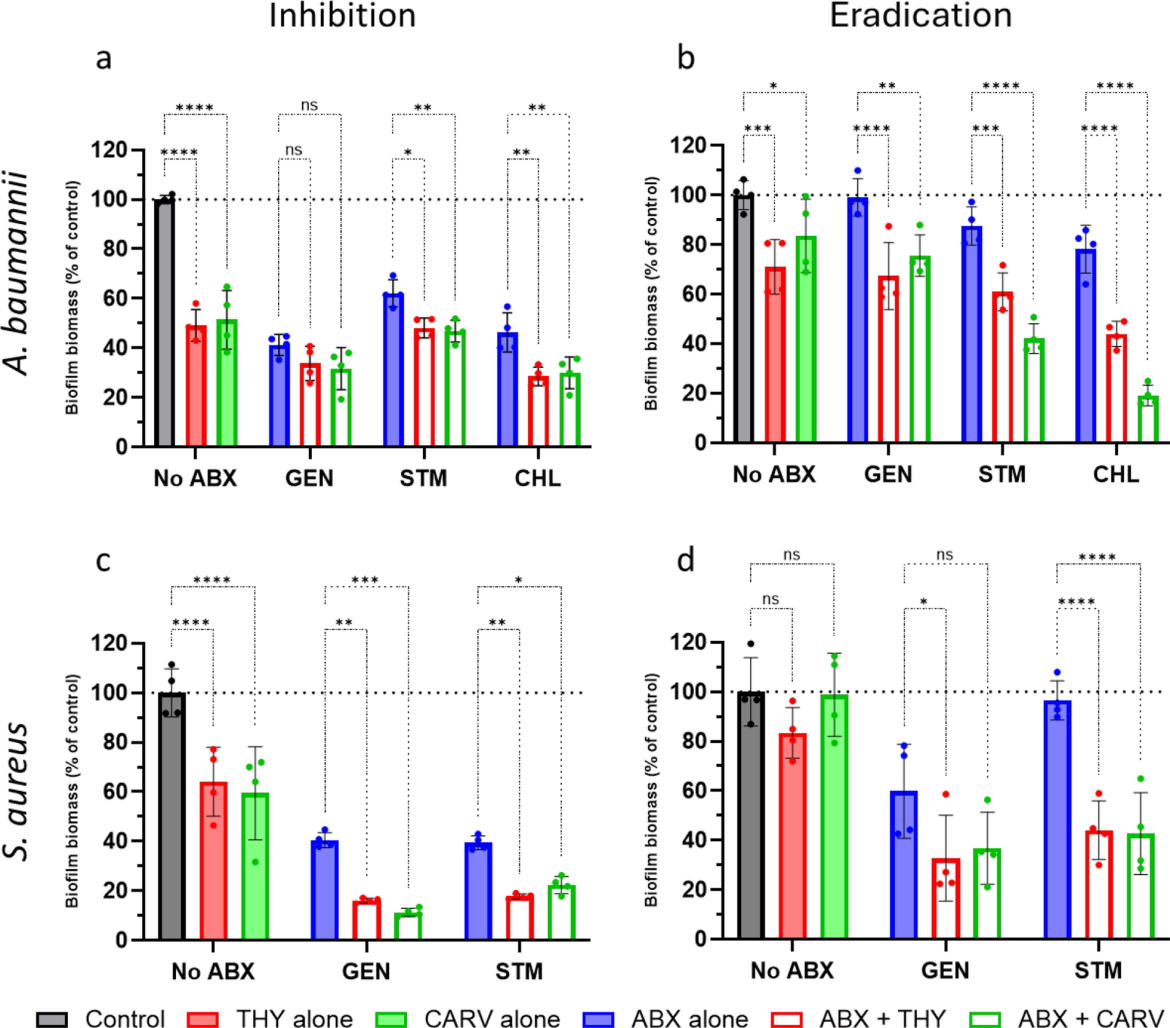
Effect of antibiotics (ABXs) alone and in combination with carvacrol (CARV) or thymol (THY) at 0.25 MIC on biofilm biomass formation and eradication in *A. baumannii* and *S. aureus*. Panels (**a**, **c**) show the biofilm biomass production (biofilm inhibition), while panels (**b**, **d**) show the eradication of preformed biofilm biomass (disruption of mature biofilms). Biofilm biomass was quantified using the crystal violet assay and is expressed as a percentage relative to the untreated control (set at 100%), representing the total biomass under each assay condition. Data are presented as mean ± standard deviation from four independent experiments. Statistical significance was determined by two-way ANOVA (****p < 0.0001, ***p < 0.001, **p < 0.01, *p < 0.05, ns = not significant)

Our results demonstrate that SACs of THY and CARV with ABXs are effective in both inhibiting biomass production and destroying preformed biofilms biomass. Figure 3 shows that certain SACs of ABXs with CARV or THY significantly reduced biofilm biomass production and effectively reduced the biomass of established biofilms, compared to ABXs and natural products alone. In *A. baumannii*, combinations of CARV with CHL reduced preformed biofilm biomass by up to 80%, compared to less than 30% achieved by CHL alone. In *S. aureus*, most combinations were more effective than ABXs alone, reaching biomass reductions of up to 70%, compared to about 40% with ABXs alone.

Previous studies have shown the efficacy of CARV and THY alone against *S. aureus* [42–44] and *A. baumannii* biofilms [45, 46]. Notably, some *A. baumannii* isolates from patients also showed significant biofilm inhibition with THY and CARV exposure [37]. A recent study [38] also investigated the effect of biofilm destruction in *S. aureus* exposed to a combination of CARV and GEN, with results very similar to ours (about 70% destruction). However, in their study, the combination of CARV and STM showed only 21.7% destruction, which yields approximately 55% biofilm destruction in our case. This discrepancy may be due to differences in the *S. aureus* strain used and methodological variations (Fig. 3d, green bars).

Different mechanisms may underlie the effects of natural products in biofilms compared to planktonic bacteria due to the unique characteristics of biofilms. For example, studies have reported that CARV can affect *S. aureus* biofilms, by potentially reducing the production of key biofilm-associated components, including extracellular polysaccharides, polysaccharide intercellular adhesin, and extracellular DNA, which would decrease biofilm density and adhesion [47]. Additionally, CARV inhibits biofilm resistance genes such as sarA and icaA, in this bacterium [43]. Another possible mechanism would be the inhibition of intercellular communication (QS), critical for biofilm formation and maintenance. By disrupting QS, the biofilm's collective resistance is weakened, increasing its susceptibility to ABXs. Other authors have demonstrated that CARV inhibits violacein and chitinase production, key QS signals in *Chromobacterium violaceum* biofilms [48]. Other proposed mechanism is reduced twitching motility due to type IV pili inhibition by THY and CARV in *A. baumannii* [45]. Numerous studies have also highlighted that the inhibition of efflux pumps in *A. baumannii* strains can also impact their biofilm-forming ability [49–51]. These pumps regulate biofilm-associated genes and actively expel QS autoinducers, antimicrobials, and metabolic intermediates, influencing QS regulation and biofilm development [52]. This activity directly and indirectly influences biofilm formation and QS regulation.

### Mechanisms of action of carvacrol and thymol

While the mechanisms of the antibiotics are well-established, understanding the contribution of natural compounds is essential for elucidating the overall mechanism of SACs. GEN and STM, aminoglycosides with broad-spectrum activity, target the 30S ribosomal subunit but differ in binding sites and effects on translation [53, 54]. CHL, an amphenicol, inhibits the 50S subunit’s peptidyl transferase, blocking peptide elongation [55]. Since all the antibiotics that exhibited synergy act intracellularly, their enhanced efficacy likely depends on increased cellular uptake or reduced efflux. To further explore how CARV and THY enhance antibiotic efficacy in SACs, we examined their impact on bacterial membranes and efflux pumps, two critical targets of natural antimicrobials that play essential roles in bacterial resistance and survival.

#### Interaction with bacterial membranes

The potential of THY and CARV to compromise bacterial envelope integrity, thereby facilitating antibiotic uptake, a critical step for the intracellular activity of GEN, STM, and CHL, was investigated by evaluating their effects on membrane potential and permeability in both species.

**Fig. 4:**
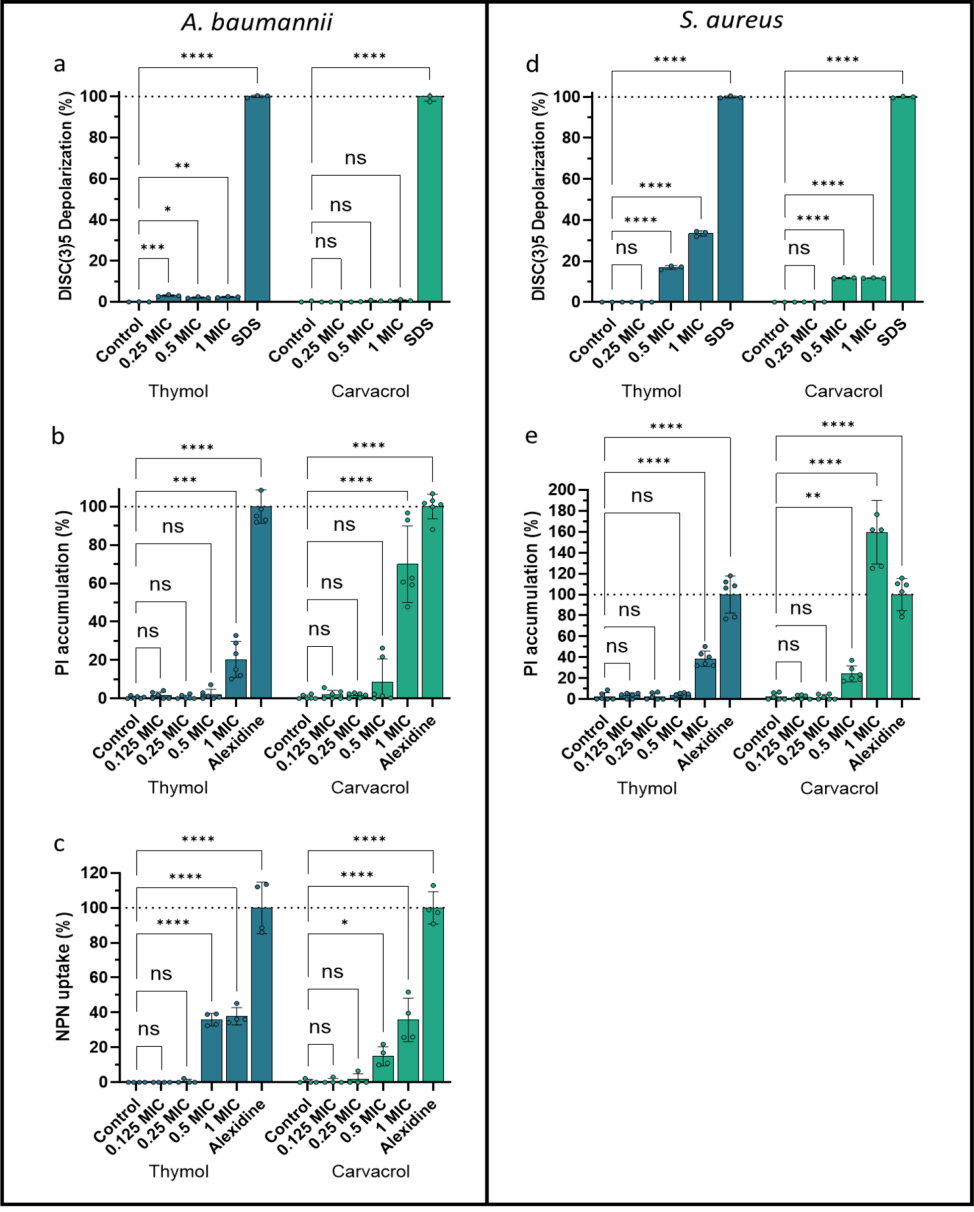
Effect of thymol and carvacrol on membrane of *A. baumannii* (**a**–**c**) and *S. aureus *(**d**, **e**). Membrane assays include: (**a**, **d**) DiSC(3)5 depolarization (expressed as % of positive control (SDS)), (**b**, **e**) inner membrane permeabilization measured by propidium iodide (PI) accumulation (expressed as % of positive control (alexidine)); and **c** outer membrane permeabilization measured by 1-*N*-phenylnaphthylamine (NPN) uptake (expressed as % of positive control (alexidine)). Statistical significance was determined by two-way ANOVA *P < 0.05; **P < 0.01; ***P < 0.001; ****P < 0.0001; ns = not significant. Error bars represent standard deviations

Figure 4 illustrates the effects of THY and CARV on membrane integrity. For *A. baumannii* (Fig. 4a–c), THY induced significant membrane depolarization at MIC or lower (Fig. 4a), while CARV showed no substantial effect on membrane potential. Both natural products disrupted the cellular membrane at MIC, with CARV showing a significantly stronger effect (*ANOVA*, p < 0.0001; Fig. 4b). Outer membrane of *A. baumannii* (Fig. 4c) was affected by both compounds at 0.5 and 1 MIC. In *S. aureus* (Fig. 4d, e), THY and CARV caused significant depolarization at their MIC and 0.5 MIC (Fig. 4d), with THY producing a higher depolarization at both concentrations (*ANOVA*, p < 0.0001). Regarding membrane disruption, both compounds induced substantial permeabilization at MIC (Fig. 4e), with CARV showing a significantly stronger effect (*ANOVA*, p < 0.0001).

These findings suggest that THY and CARV disrupt membrane integrity in both Gram-positive and Gram-negative bacteria, although their efficiencies differ. The observed actions can be attributed to the physicochemical properties of these natural products. Both CARV and THY are small molecules, with a molecular weight of 150.22 g/mol. Additionally, they are highly lipophilic, with Log Kow values of 3.43 and 3.30, respectively. These properties facilitate their diffusion through lipid membranes and allow them to embed easily into the phospholipid bilayer of bacterial membranes, altering the membrane's structure and affecting ionic gradients responsible for the membrane potential. Their lipophilicity promotes accumulation in the hydrophobic regions of the membrane, leading to destabilizing effects at the molecular level that can disrupt charge distribution or impair ion pumps and channels. This results in the leakage of protons and cations (K⁺ and Na⁺), contributing to membrane depolarization. Aminoglycosides, such as GEN and STM, activity rely significantly on active uptake into bacterial cells. This process is slow and depends on the bacterial membrane's electrochemical gradient. This gradient facilitates the active transport of aminoglycosides through specific channels or via facilitated diffusion [56]. The depolarizing effect induced by THY and CARV may facilitate aminoglycoside entry by increasing membrane permeability, thereby improving their intracellular access and enhancing their efficacy [15].

Moreover, both compounds are weak acids with a pKa of ~ 10.5, predominantly existing in a neutral form at physiological pH, facilitating membrane penetration. The phenolic hydroxyl group enables specific interactions with membrane lipids and transmembrane proteins, leading to the disruption of their structural integrity. This disruption causes the leakage of essential solutes and the entry of external substances, ultimately destabilizing the microorganism and potentially resulting in cell lysis. Indeed, our findings confirm that both THY and CARV can damage the cellular membrane of *A. baumannii* and *S. aureus*, as well as the outer membrane of *A. baumannii*'s cell wall at MIC concentrations. The structural differences between the Gram-positive cell wall of *S. aureus* and the Gram-negative wall of *A. baumannii* do not seem to hinder the effectiveness of both natural products, as both at MIC concentration, are able to disrupt the outer membrane of *A. baumannii* (Fig. 4c), penetrating the thin peptidoglycan layer to also affect the inner membrane (Fig. 4b). The minor differences between the compounds may stem from the higher hydrophobicity of CARV compared to THY, which facilitates its penetration through the bacterial membrane [57]. However, as this effect appears to occur only at high concentrations, it may explain their direct antimicrobial activity, but the synergistic effect with antibiotics does not seem to rely on this mechanism.

Our results align with microscopy studies showing that *S. aureus* exposed to CAR and THY, revealed deformed cells with projections of cellular material [58, 59], and alterations in membrane fatty acid profiles [57, 59], further affecting pH homeostasis and the equilibrium of inorganic ions [60]. Consistent with a previous study [61] that used a similar technique to determine that the membrane integrity of *S. aureus* ATCC 25923 treated with CARV at half of the MIC, a 50% disruption was observed, compared to the 24.3% observed in our study. The discrepancies may be due to slightly different technique and the different ATTC strain. Furthermore, we tested a broader range of CARV MICs (from 0.125 to 1 MIC), which allowed us to observe a progressive increase in membrane disruption, reaching over 100%. Other techniques for detecting cell membrane damage, such as the detection of the endogenous β-galactosidase enzyme, also revealed that CARV is capable of disrupting the membrane of *S. aureus* [59]. Studies also confirm THY's ability to damage bacterial membranes. For instance, Li et al. observed that, in *S. aureus*, after 6 h of exposure to 500 μg/mL, the rate of cell membrane damage increased from 0.26 to 7.82% [62]. THY also disrupts membranes in *Salmonella typhimurium, E. coli*, and *Bacillus subtilis* [63, 64].

#### Efflux pump assay

Given the critical role of efflux pumps in multidrug resistance and biofilm development, assessing their inhibition is essential for understanding the mechanism of action of antimicrobial agents. Therefore, the potential of THY and CARV to interfere with these systems was evaluated, as their inhibition could enhance intracellular antibiotic accumulation and restore ABX efficacy.

**Fig. 5:**
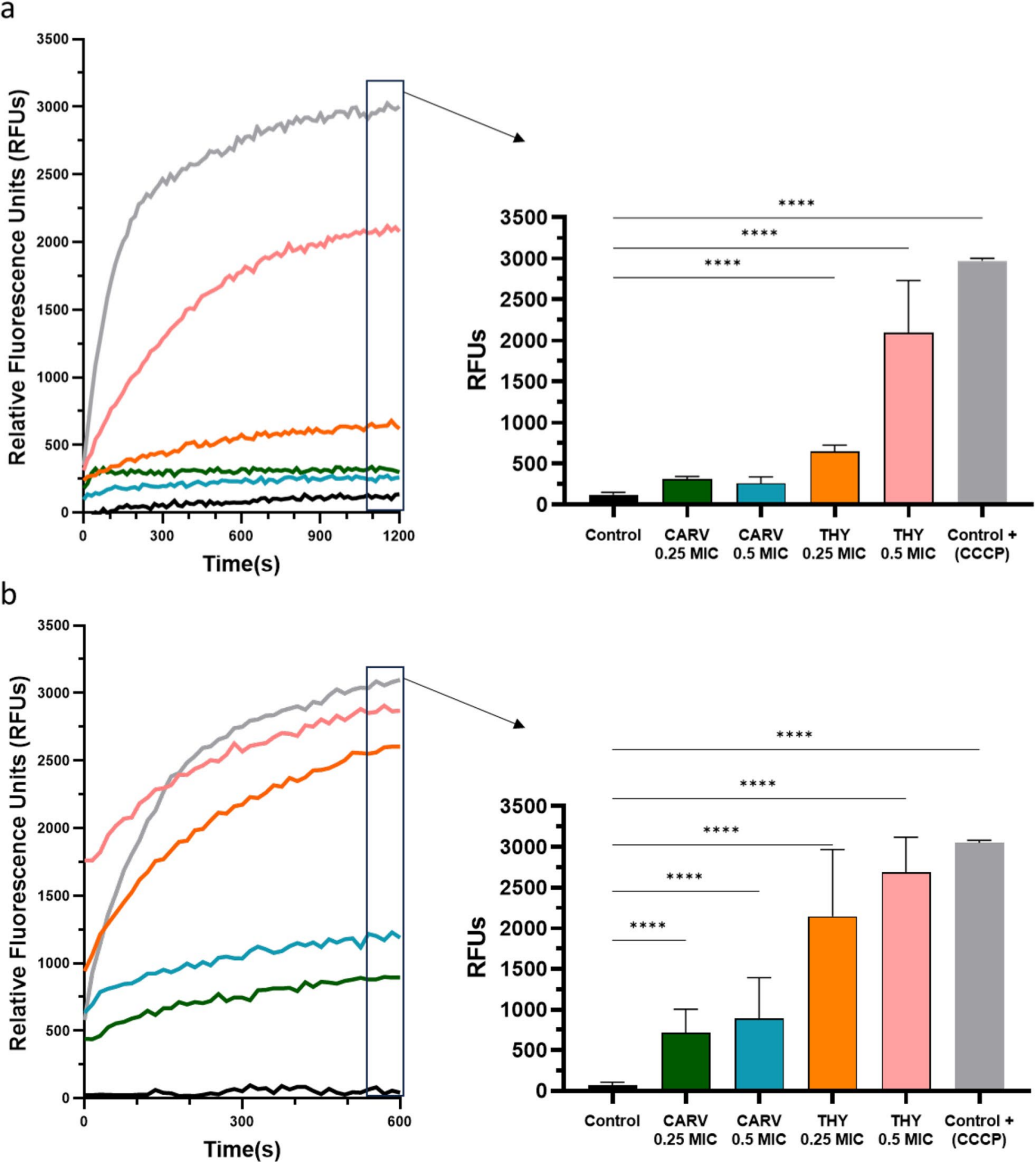
Active efflux kinetics of ethidium bromide in *A. baumannii* under two conditions: **a** acute exposure to two concentrations of thymol (THY) and carvacrol (CARV), and **b** pre-exposure of *A. baumannii* to the compounds for 6 h. Results are measured as relative fluorescence units (RFUs). Carbonyl cyanide m-chlorophenyl hydrazone (CCCP) was used as the positive control. Statistical significance was determined by two-way ANOVA: ****P < 0.0001. Error bars represent standard deviations

Figure 5 presents the active efflux kinetics of ethidium bromide (EtBr) in *A. baumannii* exposed to THY and CARV. Acute exposure to THY, at 0.5 and 0.25 MIC, significantly inhibited the efflux pumps, as evidenced by a substantial increase in intracellular EtBr fluorescence (Fig. 5a). CARV showed no significant effect under the same conditions. However, after 6 h pre-exposure (Fig. 5b), both compounds significantly inhibited efflux pump activity. THY maintained strong inhibition, while CARV showed a delayed but significant effect, suggesting that CARV requires extended exposure to interact effectively with efflux systems.

Our results support the hypothesis that THY and CARV act as efflux pump inhibitors at sub-MIC concentrations, increasing intracellular ABX accumulation thus enhancing its efficacy. Similarly, other studies have shown that THY and CARV inhibit efflux pumps in *S. aureus* strains [65, 66]. This mechanism has also been proposed as a reason why THY and CARV enhance the activity of ABXs such as tetracycline against *S. aureus* [67].

Although THY and CARV are isomers, their antimicrobial activity differs due to variations in functional group positioning, polarity, and membrane interactions. Both compounds have a phenolic hydroxyl group (−OH), but in CARV, the -OH is in the ortho position relative to the methyl group, while in THY, it is in the para position [68, 69]. These structural differences may affect how they interact with molecular targets [70].

Considering all the results, the direct antimicrobial activity of THY and CARV is likely linked to their ability to disrupt bacterial membranes. However, the adjuvant activity may instead result from their effects on membrane depolarization (in some cases) and efflux pump inhibition, allowing ABXs that rely on intracellular entry for their action to penetrate more easily and at higher concentrations.

### Effectiveness of synergistic combinations of thymol and carvacrol in preventing antibiotic resistance

Given the rise of MDR pathogens, it's essential to assess whether THY and CARV, beyond their potential synergistic antimicrobial effects, could also act as protective agents capable of reducing or preventing the development of antibiotic resistance by limiting bacterial adaptation in *S. aureus* and *A. baumannii*, both under prolonged exposure and short-term treatment.

Both *A. baumannii* and *S. aureus* are urgent threats that require immediate intervention to curb resistance development. *A. baumannii*, prevalent in hospital settings, causes severe infections like ventilator-associated pneumonia, wound infections, bacteremia, and urinary tract infections, with high mortality rates in critically ill patients. Different strains employ multiple mechanisms, such as efflux pumps and carbapenemases to resist even last-resort ABXs like carbapenems. Similarly, *S. aureus* causes infections ranging from superficial skin conditions to severe illnesses like pneumonia and bacteremia, with methicillin-resistant strains (MRSA) limiting therapeutic options and increasing mortality rates.

While serial passage and frequency selection assays provide complementary insights into the long- and short-term dynamics of resistance emergence, ROS accumulation, and ATP quantification offer mechanistic understanding of the physiological stress and metabolic disruptions induced by the treatments. Together, these approaches enable a comprehensive evaluation of both the evolutionary and cellular responses to SACs.

#### Resistance selection by serial passage

**Fig. 6:**
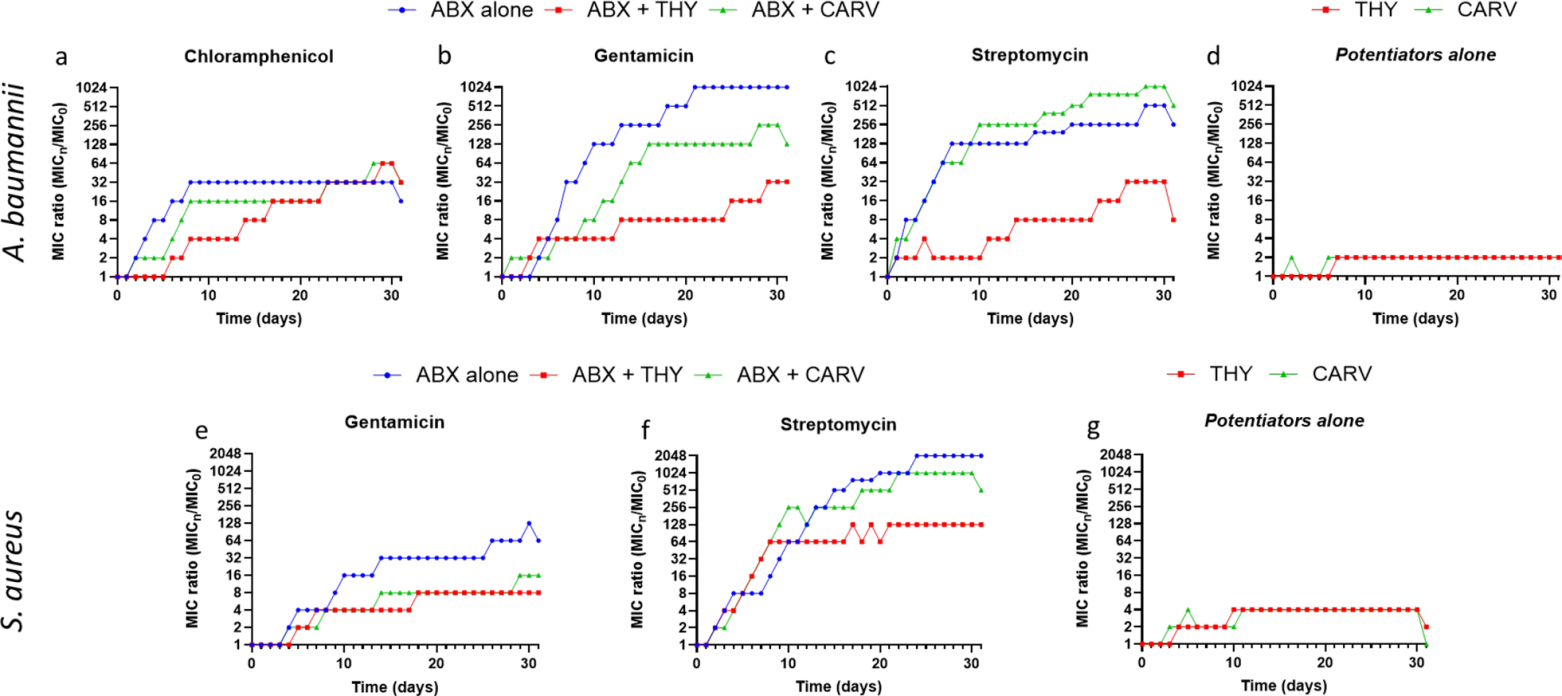
Effect of antibiotics (ABXs) and natural compounds on resistance selection (Serial passage) in *A. baumannii* (**a**–**d**) and *S. aureus* (**e**–**g**). **a**–**c** and **e**, **f** illustrate the evolution of MICs for ABXs alone or in combination with thymol (THY) or carvacrol (CARV). **d** and **g** show the MIC progression for THY and CARV alone. MIC₀ represents the initial MIC value at the beginning of the experiment, and MICₙ indicates the MIC measured after *n* days of exposure. The MIC ratio (MICₙ/MIC₀) reflects the fold increase in resistance relative to baseline and provides a quantitative measure of resistance development over time. Higher ratios indicate greater degree of resistance selection

Figure 6 shows resistance development in *A. baumannii* and *S. aureus* when exposed to ABXs, natural products, and SACs. Natural products alone did not cause significant resistance (Fig. 6d, g), whereas ABXs alone led to rapid MIC increase. *A. baumannii* exhibited a 1024-fold MIC increase for GEN and 512-fold for STM (Fig. 6b, c, blue line). In *S. aureus*, GEN MIC increased 128-fold, and STM resistance rose 2048-fold (Fig. 6e, f). SACs with THY (red line) limited MIC increases in *A. baumannii* to 32-fold for both aminoglycosides, while CARV (green line) was less effective. In *S. aureus*, THY mitigated GTM resistance to an eightfold MIC ratio increase and mitigated STM resistance to 128-fold increase, with CARV showing similar but slightly weaker effects. CHL induced slower resistance in *A. baumannii*, likely due to a different mechanism (Fig. 6a), with THY and CARV only delaying resistance.

#### Frequency of resistance (FoR)

**Fig. 7:**
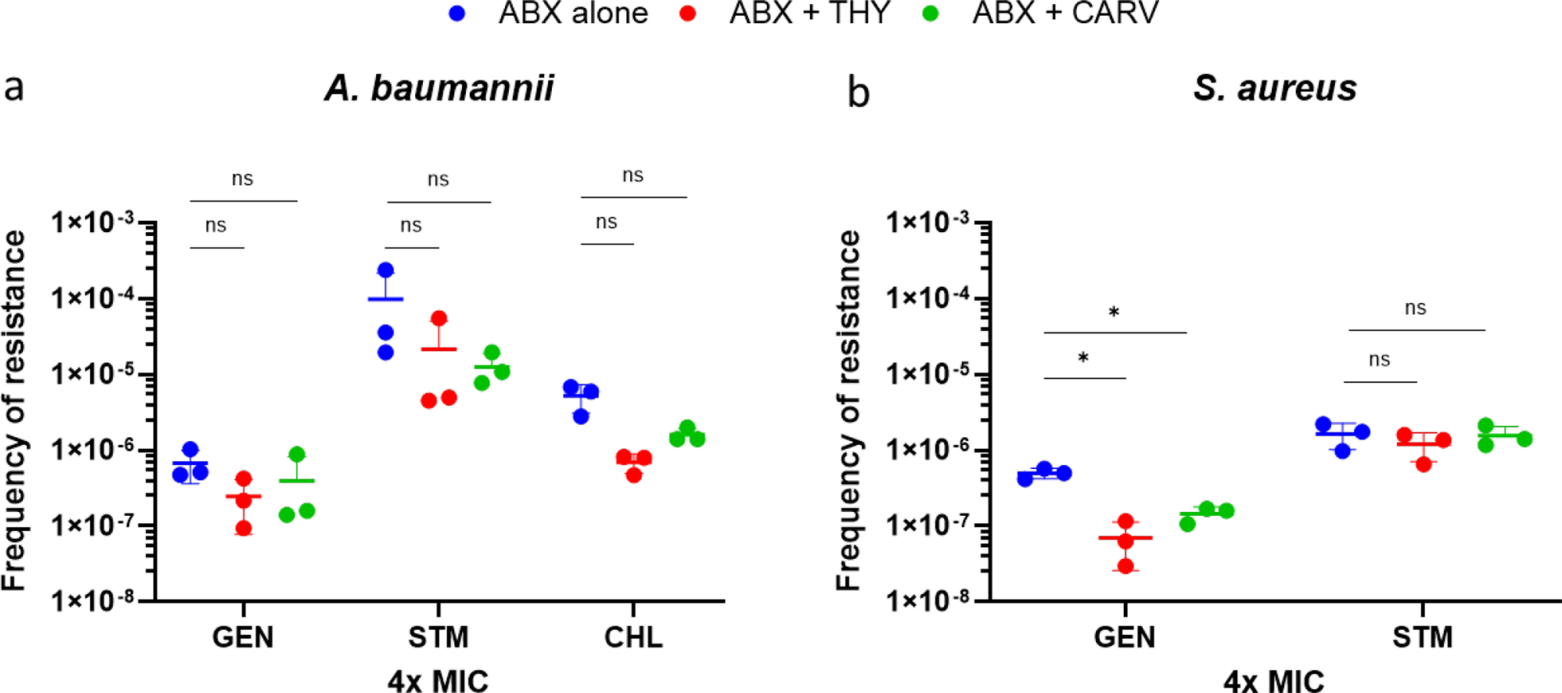
Frequency of resistance to gentamicin (GEN), streptomycin (STM) and chloramphenicol (CHL) after 48 h of incubation at 4× MIC (alone or in combination with thymol (THY) and carvacrol (CARV)) in **a** *A. baumannii* **b** and *S. aureus.* Experiments were performed in biological triplicates*.*Statistical significance was determined by t-test

FoR to ABXs was also assessed in *A. baumannii* and *S. aureus* treated with ABXs alone and combined with sub-MIC of THY or CARV (Fig. 7). The only significant reduction in resistance was observed in the combinations with GTM against *S. aureus*. THY and CARV alone showed no growth, indicating no single-step resistance generation by this natural compounds.

#### Effect of aminoglycosides on enzyme activity, ROS and ATP

We examined ROS and ATP levels in *A. baumannii* and *S. aureus* exposed to GEN and STM, alone and in combination with THY and CARV, in both original and lab-generated resistant strains to determine whether THY and CARV act as 'resistance generation protectors' by modulating bacterial oxidative stress and metabolism.

**Fig. 8:**
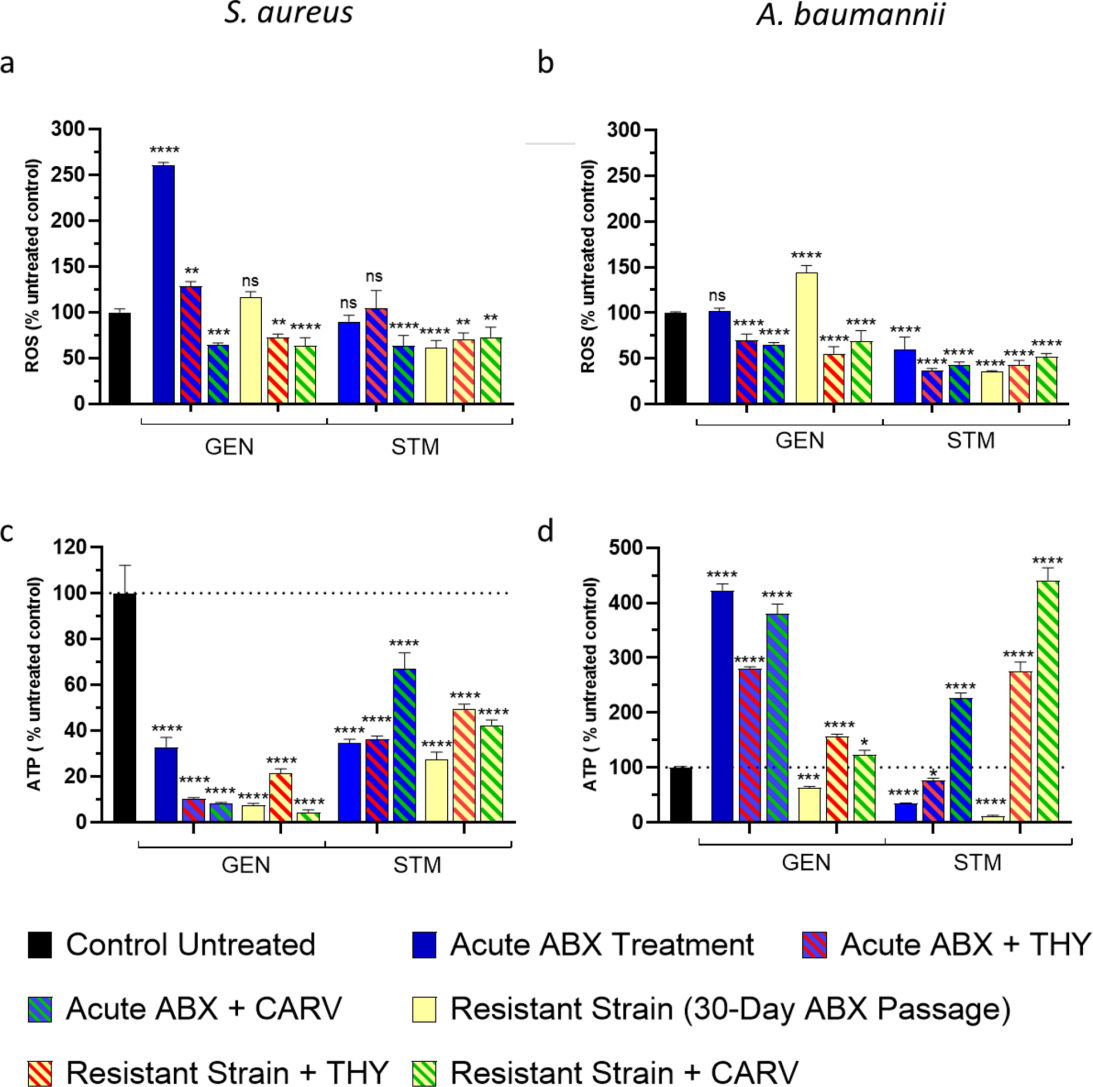
Effects of gentamicin (GEN) and streptomycin (STM) treatments on ROS and ATP levels in *S. aureus* and *A. baumannii*, including GEN- and STM-resistant strains generated via serial passage. ROS levels in *S. aureus* (**a**) and *A. baumannii* (**b**). ATP levels in *S. aureus* (**c**) and *A. baumannii* (**d**). Experimental groups include untreated control, acute exposure to GEN or STM alone and GEN or STM combined with thymol (THY) or carvacrol (CARV), in both parent and serial passage generated resistant strains. Results are presented as mean ± standard deviation (SD). Statistical significance was determined using two way Anova; *p < 0.05, **p < 0.01, ***p < 0.001, ****p < 0.0001

Acute GEN exposure raised ROS in *S. aureus* (Fig. 8a), but resistant strains showed ROS levels similar to untreated controls. THY and CARV reduced ROS, in both strains. STM did not induce ROS, but resistant strains showed reduced levels. In *A. baumannii* (Fig. 8b), acute exposure to GEN or STM did not significantly increase ROS levels; however, elevated ROS production was observed in GEN-resistant strain. Figure 8c, d reveals acute and chronic STM/GEN exposure reduced ATP in *S. aureus*, with chronic exposure causing greater depletion. In *A. baumannii*, acute GEN increased ATP, but levels normalized in resistant strains, while STM reduced ATP immediately, with chronic exposure amplifying this effect.

#### Underlying mechanisms of resistance prevention by THY and CARV as antibiotic adjuvants

All these findings highlight a key advantage of using THY and CARV as adjuvants: they reduce the in vitro emergence of antibiotic resistance in *S. aureus* and *A. baumannii* when combined with conventional antibiotics. To the best of our knowledge, this is the first report demonstrating that SACs involving these natural compounds can mitigate resistance development. Notably, few studies have explored resistance development in combinations of plant-derived natural products and commercial ABXs. For instance, it has been reported that *Aloe vera* combined with ceftiofur or cloxacillin delayed chromosomal resistance in S. *aureus* [71].

As demonstrated (Fig. 6d, g), in vitro resistance, in our selected strains, arises exclusively from ABXs, as natural products like THY and CARV did not induce resistance. This observation aligns with previous reports on other natural products [72] and CARV's effects on *S. aureus* [44]. This lack of resistance generation is likely due to the lower selective pressure, unlike conventional ABXs that target specific bacterial pathways, natural products often act on multiple bacterial targets, reducing the likelihood of selecting resistant mutants [73]. Moreover, our findings indicate that SACs containing CARV, and especially THY, can prevent the emergence of resistance induced by aminoglycosides like GEN and STM. This increase may be due to the ability of *A. baumannii* to overexpress efflux pumps when exposed to these antibiotics [74].

THY and CARV inhibit efflux pumps in *A. baumannii* (Fig. 5). This inhibition could also indirectly impact resistance development as efflux pumps enable bacteria to survive sublethal ABX doses, creating conditions that favour the selection of resistant mutants. Inhibition of efflux pumps may also indirectly influence other bacterial defense mechanisms, such as reducing the expression of resistance-associated genes. Efflux pumps are regulated by complex genetic systems that coordinate multiple resistance pathways [75, 76]. For example, the AdeABC efflux pump in our strain is controlled by the AdeRS two-component system, which play critical roles in ABX resistance and bacterial defense [77]. It has been demonstrated that inhibiting these regulators impacts in the global regulation of resistance- and virulence-associated genes, thereby weakening *A. baumannii’s* defense mechanisms [49].

This protective role of THY and CARV in preventing the emergence of ABX resistance may be linked to their ability to modulate oxidative stress in bacteria. ROS have been increasingly recognized as key players in the development of ABX resistance. Studies have shown that sublethal levels of ABXs can induce oxidative stress, leading to increased ROS production, which, in turn, promotes mutagenesis and facilitates the emergence of resistance [78]. Specifically, bactericidal ABXs such as aminoglycosides generate ROS through disruptions in bacterial respiration and metabolic activity, causing oxidative damage to DNA, proteins, and lipids [79]. This damage triggers the bacterial SOS response, a regulatory network that activates DNA repair mechanisms but also induces error-prone polymerases, which contribute to an elevated mutation rate [80]. Interestingly, mutations that arise in response to oxidative stress often target genes associated with resistance mechanisms, such as efflux pumps and porins, further driving the development of ABX resistance [81]. In addition to DNA repair, oxidative stress activates other bacterial stress responses, such as the SoxRS and OxyR systems. These pathways affect indirectly to various mechanisms, including efflux pump expression and biofilm production, which enhances ABX tolerance. Additionally, recent studies have highlighted that impaired ROS scavenging pathways, due to genetic mutations or oxidative damage, exacerbate bacterial susceptibility to oxidative stress and increase the likelihood of resistance mutations [82].

Given this context, we hypothesized that THY and CARV might protect against resistance development to ABXs by modulating oxidative stress. Notably, SACs significantly reduced ROS in *S. aureus* (Fig. 8a), supporting, along with our serial passage results (Fig. 6), the role of THY and CARV as protective agents in preventing resistance by mitigating oxidative stress. Given that oxidative stress has been linked to the activation of bacterial stress responses that promote resistance mechanisms, reducing ROS levels could help maintain cellular homeostasis and alleviate selective pressures that will favour resistance.

In the case of *A. baumannii*, the lack of oxidative stress response could be attributed to the robust mechanisms that this bacteria employs to resist oxidative damage, such as efficient antioxidant systems and the ability to modulate the expression of oxidative stress-related genes [83]. This is consistent with reports that MDR strains induce ROS production in macrophages while simultaneously upregulating catalase activity, enabling them to resist oxidative stress [84]. Likewise, *A. baumannii*, a catalase-positive bacterium, encodes catalase through the katE/katG genes, which further supports its ability to withstand oxidative damage. Nevertheless, a significant increase in ROS levels was observed in the lab-generated GEN-resistant strain. This finding supports the idea that mutations conferring resistance can impair antioxidant pathways reducing the bacterium's ability to neutralize accumulated ROS. As a result, resistant strains may exhibit higher ROS production compared to those exposed acutely to the ABX, a phenomenon previously observed in *E. coli* [81]. This phenomenon highlights the trade-off between ABX resistance and the ability to manage oxidative stress effectively. The application of THY and CARV as adjuvants significantly reduced ROS production in most cases suggesting their potential role in restoring oxidative balance and counteracting resistance mechanisms. These natural products may neutralize ROS directly through their antioxidant activity [65] or they may also modulate bacterial metabolic pathways, indirectly reducing ROS production, for instance, by stabilizing the electron transport chain or improving metabolic efficiency.

The differential effects of aminoglycoside exposure on ATP production in *S. aureus* and *A. baumannii* reveal intriguing insights into bacterial energy metabolism and its connection to antibiotic resistance. In *S. aureus*, the significant reduction in ATP levels following both acute and chronic exposure to both ABXs suggests profound metabolic disruptions. Chronic exposure exacerbates this effect, potentially reflecting long-term metabolic adaptation or damage. This reduction in ATP availability could impair the bacteria's ability to sustain vital resistance mechanisms, such as efflux pump activity and DNA repair pathways. Interestingly, the synergistic combinations of THY and CARV further amplified ATP depletion, particularly in *S. aureus* (Fig. 8c). A recently published study [82] underscores the pivotal role of central carbon metabolism and energy production in bacterial resistance. The authors demonstrated that decreased ATP production significantly impacts resistance development, particularly in *S. aureus* under GEN pressure. Moreover, GEN-resistant *S. aureus* strains were found to exhibit reduced expression of proteins involved in ATP synthesis and central carbon metabolism, including ATP synthase subunits and glycolytic enzymes. This downregulation indicates that resistance mechanisms may involve metabolic reprogramming to reduce energy demands. In *A. baumannii *(Fig. 8d), the acute exposure to GEN uniquely caused a significant transient increase in ATP levels, possibly reflecting an initial overactivation of metabolic pathways or oxidative phosphorylation. However, chronic exposure led to normalization or reduction of ATP levels, suggesting adaptive metabolic downregulation, like in *S. aureus*.

Therefore, SACs combining antibiotics with THY and CARV offer multiple approaches to preventing resistance: they mitigate oxidative stress, deplete ATP, and inhibit efflux pumps, collectively disrupting bacterial metabolic balance and resistance mechanisms. This presents a compelling strategy for combating multidrug-resistant pathogens.

**Fig. 9:**
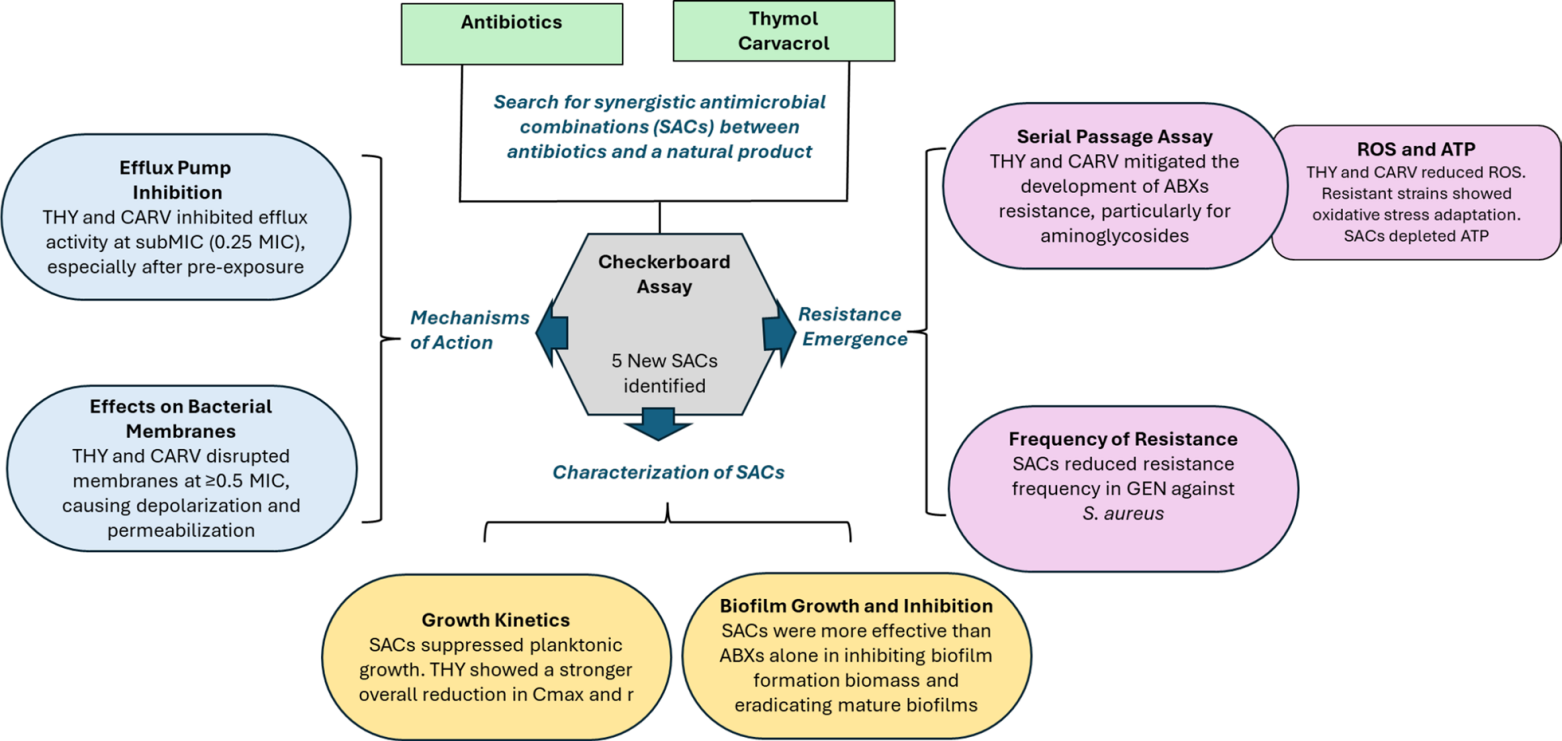
Overview of the experimental workflow, key findings, and proposed mechanisms of action for the synergistic antimicrobial combinations (SACs) of thymol (THY) and carvacrol (CARV) with antibiotics against *S. aureus* and A*. baumannii*. These findings highlight the potential of SACs to enhance antibiotic efficacy and prevent resistance, positioning them as promising candidates for alternative antimicrobial therapies. ABXs, antibiotics; MIC, minimum inhibitory concentration; Cmax, maximum population density; ROS, reactive oxygen species; GEN, gentamicin

Finally, to provide an integrated and intuitive visualization of the findings presented in this study, a summary diagram is included in Fig. 9.

## Conclusions

This study explores potential new SACs of THY and CARV with CHL, GEN and STM against *S. aureus* and *A. baumannii*. The SACs demonstrated good antimicrobial efficacy, completely inhibiting planktonic bacterial growth and outperforming ABXs alone, including superior biofilm inhibition and destruction. Notably, both THY and CARV acted as resistance-protective agents at subMIC concentrations, preventing the emergence of resistance when combined with aminoglycosides. For the first time, we also comprehensively studied the mechanisms of action, revealing how THY and CARV induce membrane depolarization, damage cell membranes, inhibit efflux pumps, modulate oxidative stress and disrupt bacterial energy metabolism, thus enhancing their antimicrobial effects and preventing resistance. These findings position SACs as promising candidates in the fight against multidrug-resistant pathogens.

## Data Availability

The datasets generated during and/or analysed during the current study are available from the corresponding author on reasonable request.
